# Solasonine induces apoptosis of the SGC‐7901 human gastric cancer cell line in vitro via the mitochondria‐mediated pathway

**DOI:** 10.1111/jcmm.17343

**Published:** 2022-05-07

**Authors:** Tian‐Chuan Li, Nai‐Jie Chen, Yun‐Ying Chen, Bing‐Jing He, Zhi‐Feng Zhou

**Affiliations:** ^1^ 74551 School of Basic Medical Sciences Fujian Medical University Fuzhou China; ^2^ Department of Integrated Traditional and Western Medicine Fujian Medical University Cancer Hospital & Fujian Provincial Cancer Hospital Fuzhou China; ^3^ Fujian Key Laboratory of Translational Cancer Medicine Fuzhou China; ^4^ Laboratory of Immuno‐Oncology Fujian Medical University Cancer Hospital & Fujian Provincial Cancer Hospital Fuzhou China

**Keywords:** apoptosis, cell cycle, endoplasmic reticulum stress pathway, gastric cancer, HPLC‐MS/MS, mitochondrial pathway, proliferation, *Solanum nigrum*, Solasonine

## Abstract

Solasonine, a steroidal glycoalkaloid isolated from the herbal plant *Solanum nigrum* Linn., has shown active against multiple human cancers; however, there is little knowledge on the activity of solasonine against gastric cancer until now. This study aimed to examine the effect of solasonine on the biological behaviours of human gastric cancer SGC‐7901 cells. The results showed that solasonine suppressed SGC‐7901 cell proliferation in a dose‐dependent manner. Solasonine treatment mainly induced the cell cycle arrest at G2 phase in SGC‐7901 cells. Treatment with solasonine resulted in significant down‐regulation of Bcl‐2 and Caspase‐3 protein expression and reduced Bax and Bcl‐xL protein expression in SGC‐7901 cells. Solasonine shows a comparable inhibitory effect on the proliferation of human gastric cancer SGC‐7901 cells with cisplatin, and solasonine induces of SGC‐7901 cell apoptosis through triggering the endoplasmic reticulum stress pathway and the mitochondrial pathway. Our data indicate that solasonine may be a promising agent for the treatment of gastric cancer.

## 1 INTRODUCTION

Gastric cancer is the fourth most common cancer and the third leading cause of cancer death worldwide.^1^ There were approximately 1.03 million new cases diagnosed with gastric cancer, and more than 782 thousand people died from this malignancy across the world in 2018.^2^ Currently, the options for treatment of gastric cancer mainly include gastrectomy, chemotherapy, radiotherapy and emerging targeted therapy.^3^, ^4^, ^5^ These treatments achieve a more than 90% 5‐year survival rate in patients with early‐stage gastric cancer; however, the prognosis remains unsatisfactory in advanced patients.^6^ Screening of and search for novel approaches for the management of gastric cancer, as a supplement to currently available treatments, is therefore of great significance to improve the prognosis of gastric cancer patients.^7^

*Solanum nigrum* Linn. is a herbal plant that frequently grows in temperate climate regions.^8^ As a traditional Chinese medicine, *S*. *nigrum* has shown antitumour, anti‐infective, antiphlogistic, febrifuge, sedative, analgesic, lipid‐lowering, hepato‐protective, antitussive and expectorant, antiviral, antimicrobial and anti‐shock actions.^9^, ^10^ Solasonine, a steroidal glycoalkaloid isolated from *S*. *nigrum*,^11^ has shown active against hepatocellular carcinoma (HCC), glioma, lung cancer and colorectal cancer.^12^, ^13^, ^14^, ^15^, ^16^ However, there is little knowledge on the activity of solasonine against gastric cancer until now.^17^, ^18^ This study was therefore designed with aims to examine the effect of solasonine on the biological behaviours of human gastric cancer SGC‐7901 cells.

## 2 MATERIALS AND METHODS

### 2.1 Drugs

The traditional Chinese herb *S*. *nigrum* was purchased from Fujian Medical University Union Hospital (Fuzhou, China). Solasonine (purity, 95% and greater; lot no. SML1141) was purchased from Sigma‐Aldrich (St. Louis, MO, USA). Cisplatin (cisplatin for injection, 5 mg/ml) was provided Jiangsu Hansoh Pharmaceutical Group Co., Ltd. (Lianyungang, China).

### 2.2 Extraction of solasonine

*Solanum nigrum* was ground into powder measuring approximately 0.1 mm^3^. Then, 50 g powder was immersed in 250 ml ethanol for 6 hours, followed by ultrasonic‐assisted extraction for 30 min. The leaching was repeated in triplicate until the leaching solution was colourless. The triplicate leaching solutions were merged together, and dried under reduced pressure distillation at 40°C to yield the ethanol extracts of *S*. *nigrum*.

*Solanum nigrum* powder (50 g) was immersed in 250 ml purified water for 2 hours, followed by boiling for 3 h. The boiling solution was filtered, and the filter residue was boiled twice in 250 ml purified water. All boiling solutions were merged together, and dried under reduced pressure distillation and vacuum freezing to yield the water extracts of *S*. *nigrum*.

The ethanol and water extracts of *S*. *nigrum* were dissolved in 30% methanol to prepare into the test sample at 50 mg/ml. The concentration of solasonine was determined using high‐performance liquid chromatography‐electrospray tandem mass spectrometry (HPLC‐MS/MS).

### 2.3 Qualitative and quantitative detection of solasonine

Solasonine standard sample was dissolved in chromatographic pure methanol to prepare into stock solutions at a concentration of 10 mg/ml, and diluted into solutions at 10, 20, 30, 40 and 50 μg/ml with 30% methanol. The test samples were dissolved in 30% methanol and prepared into different concentrations for the subsequent detections. The qualitative and quantitative detection of solasonine was performed using HPLC‐MS/MS with an YMC‐Triart C18 column (2.1 × 100 mm, 1.9 μm) at a column temperature of 35°C. The gradient elution programme comprised 30% mobile phase A (99.9% methanol and 0.1% formic acid) and 70% mobile phase B (99.9% deionized water and 0.1% formic acid) for 1 min at a flow rate of 0.35 ml/min. The gradient gradually increased to 80% mobile phase A until 8 min and was maintained at 80% until 10 min. The loading volume was 2.0 μl.

MS/MS analysis was performed with an electrospray ionization (ESI) probe operated at a spray voltage of 2.5 kV, cone gas with a flow rate of 50 L/h, an ionization source temperature of 150°C, desolvation gas with a flow rate of 13 L/h, sweeping gas with a flow rate of 1 L/h and capillary temperature of 320°C. MS/MS scanning was conducted in a multiple‐reaction monitoring (MRM) mode at a resolution of 35,000, parent ion of 884.6 m/z, secondary quantitative ion of 85.00 m/z and qualitative ion of 866.55 m/z. Then, the standard curve was plotted.

### 2.4 Cell line and culture

Human gastric cell line SGC‐7901 was provided by the central laboratory of Zhongshan Hospital Affiliated to Xiamen University (Xiamen, China) and maintained in liquid nitrogen at Fujian Provincial Cancer Hospital (Fuzhou, China). Frozen SGC‐7901 cells were rapidly thawed in a water bath at 37°C. Then, cells were incubated in RPMI 1640 medium (Hyclone; Logan, UT, USA) supplemented with 10% foetal bovine serum (Gibco; Grand Island, NY, USA), 100 U penicillin and 100 g/ml streptomycin (Shanghai Basalmedia Technologies Co., Ltd.; Shanghai, China) at 37°C containing 5% CO_2_ for 48 hours. After cells grew to approximately 80% confluence, the medium and suspended cells were removed, and cells adherent to the petri dish wall were washed with 37°C pre‐warmed sterile PBS (Shanghai Basalmedia Technologies Co., Ltd.; Shanghai, China), added with 1 ml 0.25% pancreatin (Hyclone; Logan, UT, USA) for 1 min digestion to allow cells to separate from the wall. Subsequently, the petri dish was added with 3 ml 37°C pre‐warmed RPMI 1640 medium, and cells were passaged at a ratio of 1:2. After two passages, cells were harvested for the subsequent experiments.

### 2.5 CCK‐8 assay

The SGC‐7901 cell proliferation was measured with a CCK‐8 assay. Briefly, SGC‐7901 cells were seeded onto 96‐well plates (Corning, Inc.; Corning, NJ, USA) at a density of 1.5 × 10^5^ cells/ml, and each well was added with 100 μL cells, and incubated at 37°C containing 5% CO_2_ for 24 hours. Then, the medium was removed, and fresh RPMI 1640 medium containing solasonine or cisplatin at final concentrations of 1, 2.5, 5, 10, 20, 30 and 50 μg/ml, with 5 replicate wells assigned for each drug concentration, while fresh medium served as a negative control. After cells were incubated at 37°C containing 5% CO_2_ for 24 h, each well was transferred with 10 μl CCK‐8 solutions (MedChemExpress LLC; Monmouth Junction, NJ, USA) and gently mixed evenly. Subsequently, cells were incubated for a further one hour, and the optical density at a wavelength of 450 nm was measured with an M1000 Pro multimode microplate reader (Tecan Group; Männedorf, Switzerland). The half‐maximal inhibitory concentration (IC_50_) of solasonine against SGC‐7901 cells was estimated.

### 2.6 Analysis of cell cycle of SGC‐7801 cells

The cells cycle of SGC‐7901 cells was analysed using flow cytometry. Briefly, SGC‐7901 cells were seeded onto 6‐well plates (Corning, Inc.; Corning, NJ, USA) at a density of 8 × 10^5^ cells/well, and each well was added with RPMI 1640 medium to 2 ml. Cells were incubated at 37°C containing 5% CO_2_ for 24 h, and then, the medium was discarded and 2 ml fresh RPMI 1640 medium containing solasonine, cisplatin or solasonine‐cisplatin combinations were added, while fresh RPMI 1640 medium served as a control. Three replicate wells assigned for each treatment. After cells were incubated at 37°C containing 5% CO_2_ for 24 h, the medium was removed and the plate wall‐adherent cells were washed once with PBS and digested with 1 ml 0.25% pancreatin for 1 min. Cells were gently separated with a pipette, transferred to 15 ml centrifugation tubes and centrifuged at 800*g* for 5 min at room temperature. The supernatant was removed, and cells were re‐suspended in 2 ml 4°C pre‐cooled PBS and centrifuged at 800*g* for 5 min at room temperature. The supernatant was discarded, and cells were re‐suspended in 1 ml ice‐water bath pre‐cooled PBS, slowly added with ‒20°C pre‐cooled absolute ethanol to yield a final concentration of 70% and incubated in water bath at 4°C for 12 hours. Subsequently, cells were centrifuged at 800*g* for 5 min at 4°C, and the supernatant was removed. Cells were washed in 1 ml 4°C pre‐cooled PBS, added with propidium (PI) staining solution (Thermo Fishier Scientific; Waltham, MA, USA) at a final concentration of 50 μg/ml, RNase A (50 μg/ml) and 0.2% Triton X‐100. Cell solutions were gently mixed evenly and incubated in darkness for 30 min at 4°C. Cell cycle analysis was done on an FC500 flow cytometer (Beckman Coulter; Brea, CA, USA), and the proportions of cells at sub‐G0 phase, G0/G1 phase, S phase and G2/M phase were estimated using the software ModFit LT version 3.3.

### 2.7 Detection of SGC‐7901 cell apoptosis

The apoptosis of SGC‐7901 cells was detected using flow cytometry. Briefly, SGC‐7901 cells were seeded onto 6‐well plates at a density of 8 × 10^5^ cells/well, and each well was added with RPMI 1640 medium to 2 ml. Cells were incubated at 37°C containing 5% CO_2_ for 24 h, and then, the medium was discarded and 2 ml fresh RPMI 1640 medium containing solasonine, cisplatin or solasonine‐cisplatin combinations were added, while fresh RPMI 1640 medium served as a control. Three replicate wells assigned for each treatment. After cells were incubated at 37°C containing 5% CO_2_ for 24 h, the medium was removed, and the plate wall‐adherent cells were washed once with PBS and digested with 1 ml 0.25% pancreatin for 1 min. Cells were gently separated with a pipette, transferred to 15 ml centrifugation tubes and centrifuged at 800*g* for 5 min at room temperature. The supernatant was removed, and cells were re‐suspended in 2 ml ice‐water bath pre‐cooled PBS, washed once with PBS, and the supernatant was discarded. Cells were re‐suspended in 195 μl Annexin V binding buffer (Thermo Fishier Scientific; Waltham, MA, USA), mixed evenly with 5 μl Annexin V‐FITC (Thermo Fishier Scientific; Waltham, MA, USA), incubated in darkness at room temperature for 10 min and centrifuged at 800*g* for 5 min at room temperature. Subsequently, cells were re‐suspended in 190 μl Annexin V binding buffer, mixed gently with 5 μl PI staining solution and incubated in ice‐water bath for 15 min in darkness. Cell apoptosis was detected on an FC500 flow cytometer, and the cell apoptotic rate was calculated using the software ModFit LT version 3.3.

### 2.8 Western blotting assay

The expression of apoptotic regulatory proteins was quantified using a Western blotting assay. Briefly, SGC‐7901 cells were seeded onto 6‐well plates at a density of 8 × 10^5^ cells/well, and each well was added with RPMI 1640 medium to 2 ml. Cells were incubated at 37°C containing 5% CO_2_ for 24 h, and then, the medium was discarded and 2 ml fresh RPMI 1640 medium containing 18 μM solasonine, 17.5 μM cisplatin or a combination of 18 μM solasonine and 17.5 μM cisplatin were added, with 3 replicate wells assigned for each treatment. After cells were incubated at 37°C containing 5% CO_2_ for 24 h, the medium was removed and the plate wall‐adherent cells were washed twice with 4°C pre‐cooled PBS. Each well was added with 200 μl protein lysis buffer (Beyotime Biotechnology; Shanghai, China), and cells were lysed and pipetted gently on ice for 10 min. Then, the lysis buffer was transferred to 1.5 ml centrifugation tubes, treated with a Vibra‐Cell™ ultrasonic processor (Sonics & Materials, Inc.; Newtown, CT, USA) for 30 s, and centrifuged at 12,000*g* for 15 min at 16°C. The supernatant was collected, and the protein concentration was quantified using a BCA assay (Beyotime Biotechnology; Shanghai, China).

Approximately 30 μg total protein was separated on 12% SDA‐PAGE and then transferred to polyvinylidene difuoride (PVDF) membrane (NEN Life Science Products; Boston, MA, USA). The blotted membrane was incubated with the specific primary mouse anti‐Bcl‐2, anti‐Bax, anti‐Caspase‐3 and anti‐GAPDH antibodies (Abcam; Cambridge, MA, USA) overnight at 4°C. Then, the membranes were washed twice in TBST for 15 min, incubated with the HRP‐conjugated secondary IgG antibody (1:5000 dilution; Abcam, Cambrige, MA, USA) for 30 min at room temperature. The membranes were washed three times in TBST, of 5 min each time, and the blots were visualized with the ECL reagent (Beyotime Biotechnology, Shanghai, China) for 1 min, then, the images were captured with the Fluor™ plus gel imaging system (Omega Bio‐Tek, Inc., Norcross, GA, USA). The band density was analysed using the software Quantity One version 4.6.7 (Bio‐Rad; Hercules, CA, USA), and the target protein expression was normalized to GADPH.

### 2.9 Statistical analysis

All data were the representatives of at least three independent experiments, and described as mean ± standard deviation (SD). Differences of means between groups were tested for statistical significance with Student's *t* test, and a *P* value of < 0.05 was considered statistically significant.

## 3 RESULTS

### 3.1 Solasonine contents in *S*. *nigrum* extracts

**FIGURE 1:**
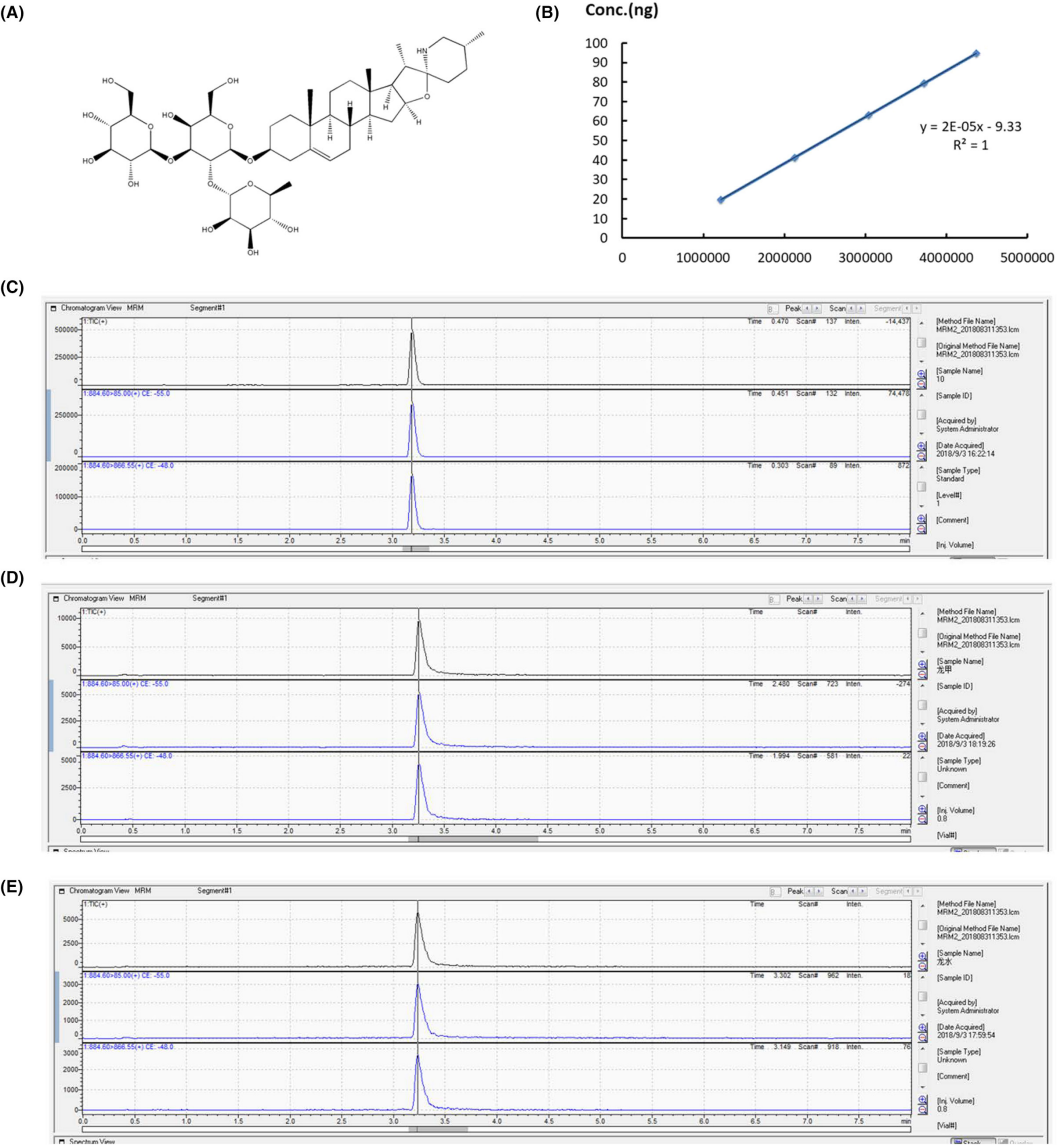
HPLC‐MS/MS analysis of solasonine. (A) molecular structure of solasonine (molecular formula of C_45_H_73_NO_16_, molecular weight of 884.06); (B) standard curve for a series of solasonine standard sample gradients as qualitatively and quantitatively assessed by MS/MS; (C) qualitative and quantitative detection of solasonine standard sample by MS/MS, with parent ion of 884.60 m/z, quantitative ion of 85.00 m/z and qualitative ion of 886.55 m/z; (D) qualitative and quantitative detection of solasonine in ethanol extracts of *Solanum nigrum*; (E) qualitative and quantitative detection of solasonine in water extracts of *Solanum nigrum*

Figure 1A shows the molecular structure of solasonine, which has a theoretical molecular weight of 884.06. MS/MS detects three isotopic ion peaks of solasonine at 883.5, 884.5 and 885.5 m/z. HPLC‐MS/MS analysis detected the optimal parent ion of 884.6 m/z, qualitative ion of 866.55 m/z and quantitative ion of 85.00 m/z, and the qualitative and quantitative detection of solasonine were established. Figure 1B shows that the correlation coefficient (*R* ^2^) for solasonine concentrations (20 to 100 ng) and ion intensity approached 1, indicating that the established HPLC‐MS/MS assay is effective for the qualitative and quantitative analyses of solasonine. The retention time, primary ion and secondary qualitative and quantitative ions were highly consistent between the solasonine in the ethanol and water extracts of *S*. *nigrum* and the standard sample of solasonine (Figure 1C, D and E). The solasonine concentration was estimated to be 0.4 mg/g in the ethanol extracts of *S*. *nigrum*, 0.28 mg/g in the water extracts of *S*. *nigrum* and 0.68 mg/g in the test sample.

### 3.2 Solasonine inhibits the proliferation of SGC‐7901 cells

**FIGURE 2:**
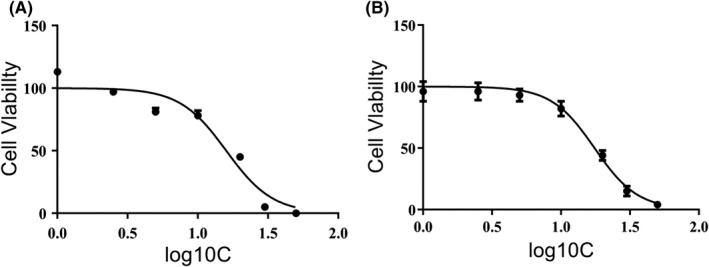
CCK‐8 assay determines the in vitro inhibition of solasonine and cisplatin on SGC‐7901 cells. (A) the 24 h cisplatin IC_50_ is 17.5 μM against SGC‐7901 cells; (B) the 24 h solasonine is 18 μM against SGC‐7901 cells

Exposure to solasonine for 24 h was found to remarkably suppress the in vitro proliferation of SGC‐7901 cells in a dose‐dependent manner (Figure 2). The 24 h IC_50_ value of solasonine was 18 μM against SGC‐7901 cells, which was similar to that of cisplatin (17.5 μM), indicating that solasonine shows a highly inhibitory activity against SGC‐7901 cell proliferation.

### 3.3 Effect of solasonine on the cell cycle of SGC‐7901 cells

**FIGURE 3:**
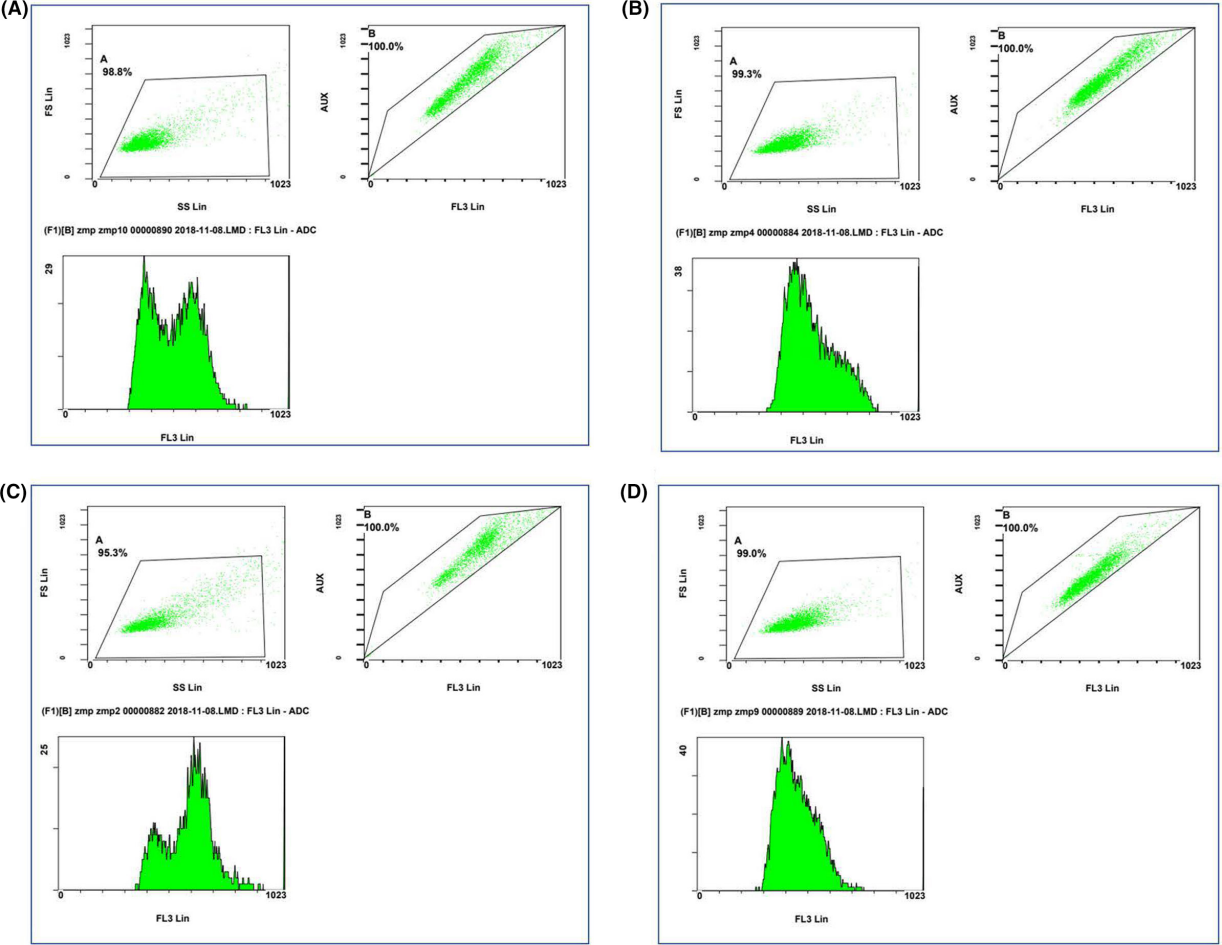
Effect of solasonine on the cell cycle of SGC‐7901 cells. (A) negative controls; (B) treatment with 17.5 μM cisplatin for 24 h; (C) treatment with 18 μM solasonine for 24 h; (D) combined treatment with 17.5 μM cisplatin and 18 μM solasonine for 24 h

Flow cytometry revealed that the SGC‐7901 cells exposed to fresh RPMI 1640 medium were evenly distributed at G1/G1, S and G2/M phases (Figure 3A), and cisplatin‐treated SGC‐7901 cells were predominantly arrested at G0/G1 phase, with a few cells at S and G2/M phases (Figure 3B), which in in agreement with cisplatin‐induced DNA replication.^19^ Following exposure to solasonine for 24 h, SGC‐7901 cells were predominantly arrested at G2 phase (Figure 3C). These data indicate that solasonine does not suppress the SGC‐7901 cell proliferation through inhibiting DNA replication, but through suppressing the diploid cell division, which is remarkably different from the mode of action of cisplatin. In addition, the combined treatment with solasonine and cisplatin resulted in cell cycle arrest at G0/G1 phase in most SGC‐7901 cells, and a few cells were detected at G2 phase (Figure 3D). These findings demonstrate that cisplatin‐induced inhibition of DNA replication may exhibit a predominant role, and solasonine may promote the cisplatin activity. Our data suggest that the solasonine‐cisplatin combination may achieve a higher therapeutic efficacy against gastric cancer.

### 3.4 Effect of solasonine on the apoptosis of SGC‐7901 cells

**FIGURE 4:**
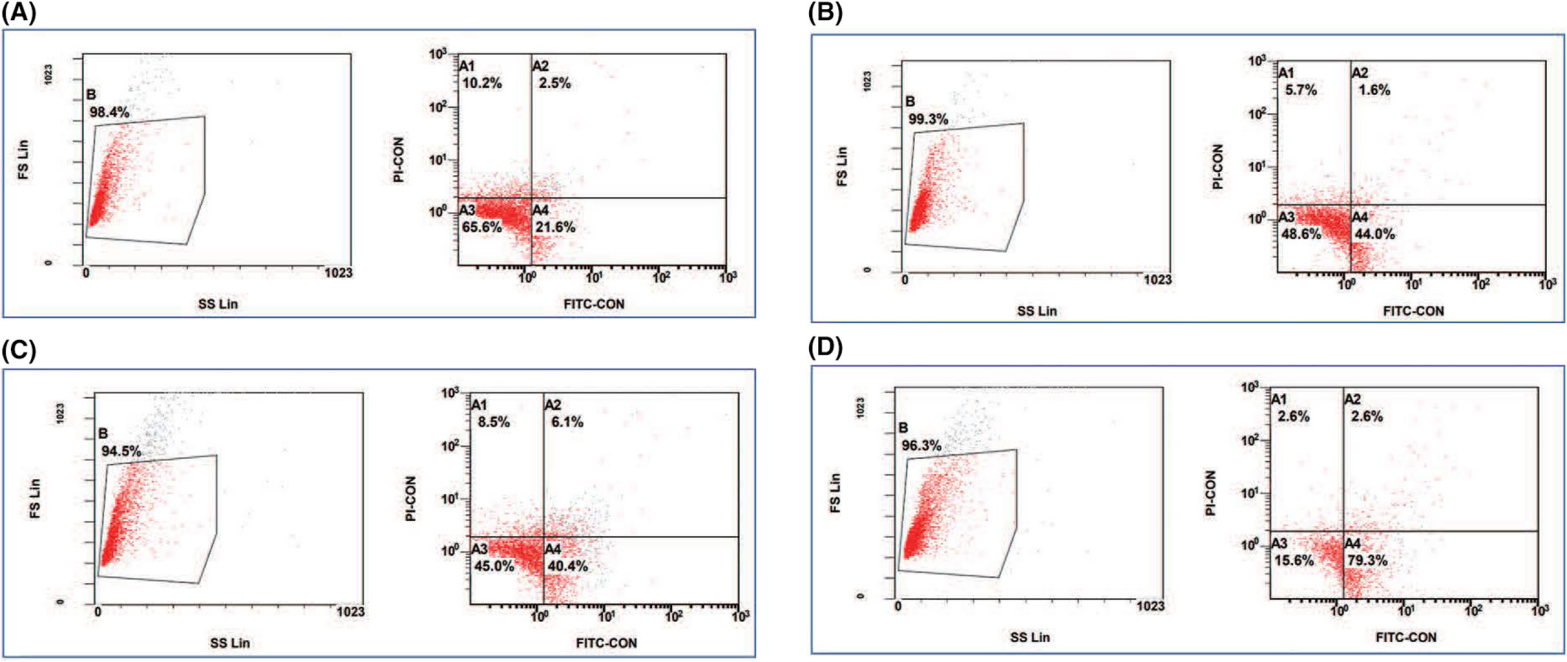
Effect of solasonine on SGC‐7901 cell apoptosis. (A) negative controls; (B) treatment with 17.5 μM cisplatin for 24 h; (C) treatment with 18 μM solasonine for 24 h; (D) combined treatment with 17.5 μM cisplatin and 18 μM solasonine for 24 h

Flow cytometry detected a 21.6% apoptotic rate in SGC‐7901 cells treated fresh RPMI 1640 medium for 24 h, indicative of normal SGC‐7901 cell morphology (Figure 4A). Following exposure to 17.5 μM cisplatin and 18 μM solasonine for 24 h, the apoptotic rates of SGC‐7901 cells were 44% and 40.4%, respectively, (Figure 4B and C), and the combined treatment with 17.5 μM cisplatin and 18 μM solasonine for 24 h resulted in a 79.3% apoptotic rate of SGC‐7901 cells, indicating the synergistic effect of the cisplatin‐solasonine combination on SGC‐7901 cell apoptosis.

### 3.5 Effect of solasonine on the expression of apoptotic regulatory proteins in SGC‐7901 cells

**FIGURE 5:**
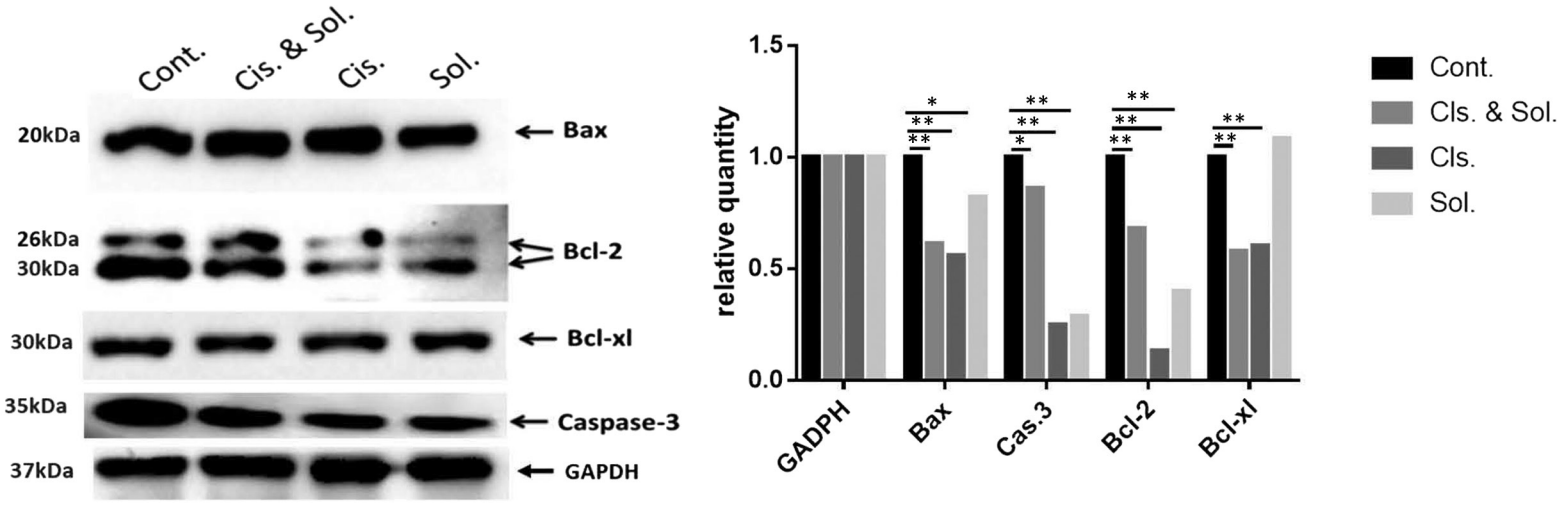
Effect of solasonine on the expression of apoptosis‐associated proteins in SGC‐7901 cells. Cont., negative controls; Cis. * Sol., combined treatment with 17.5 μM cisplatin and 18 μM solasonine for 24 h; Cis., treatment with 17.5 μM cisplatin for 24 h; Sol., treatment with 18 μM solasonine for 24 h. * *p* < 0.05, ** *p* < 0.01 vs. negative controls

Western blotting assay detected a remarkable reduction in Bcl‐2, Caspase‐3, Bax and Bcl‐xL protein expression in SGC‐7901 cells following treatment with cisplatin and solasonine for 24 h; however, the down‐regulation was more significant in SGC‐7901 cells caused by treatment with cisplatin alone. In addition, treatment with solasonine alone resulted in significant down‐regulation of Bcl‐2 and Caspase‐3 protein expression and reduced Bax and Bcl‐xL protein expression in SGC‐7901 cells as compared with the RPMI 1640 medium treatment (Figure 5). Our data demonstrate that treatment with solasonine, cisplatin alone or in combination induces SGC‐7901 cell apoptosis via the mitochondrial apoptotic pathway.

## 4 DISCUSSION

As a traditional Chinese medicinal herb, *S*. *nigrum* is widespread in China^8^ and has shown a wide range of pharmacological actions.^9^, ^10^ In this study, a HPLC‐MS/MS‐based assay was established for the qualitative and quantitative assessment of solasonine, and the solosonine concentration was approximate 0.68 mg/g in the traditional Chinese medicinal herb *S*. *nigrum*. In this study, quantitative and qualitative analyses showed that solasonine content was not high in the medicinal herb *Solanum nigrum*, which may be associated with the place of production, time of collection and freshness degree of *Solanum nigrum* decoction pieces. *Solanum nigrum* has shown a remarkable efficacy for clinical treatment of gastric cancer, and the common prescription is 50 g fresh *Solanum nigrum* per dose or 30 g dried *Solanum nigrum* per dose.

Recently, solasonine, a steroidal glycoalkaloid isolated from the medicinal plant *S*. *nigrum*, has been found to be active against multiple human cancers.^12^, ^13^, ^14^, ^15^, ^16^ A recent study showed that solasonine significantly suppressed proliferation of HCC HepG2 and HepRG cells in vitro, and suppressed HepG2 cell‐derived tumour volume and weight, and inhibited HepG2 cell migration and invasion in a mouse xenograft model, and metabolomics analysis revealed that solasonine promoted ferroptosis of HCC cells via GPX4‐induced destruction of the glutathione redox system.^12^ In addition, solasonine was found to suppress the proliferation, migration and colony formation of glioma U87 MG, U251 and U118 MG cells through inhibiting inflammatory signalling pathway^14^ and suppress the proliferation of lung cancer H446 cells through up‐regulating pro‐apoptotic protein expression and down‐regulating anti‐apoptotic protein expression.^15^ Wu et al. found that solasonine enhanced the sensitivity of oesophageal cancer EC9706 and KYSE30 cells to 5‐flurouracil (5‐FU) and cisplatin through inducing miR‐138 expression,^20^ and Hasanain and colleagues reported that solasonine induced autophagy to exert anti‐proliferative activity non‐small‐cell lung cancer A549 cells, human breast cancer MCF‐7 cells and human prostate cancer DU145 cells by triggering endoplasmic reticulum stress and inhibiting Akt/mTOR signalling.^21^ Moreover, solasonine was found to suppress the RL95‐2 oestrogen receptor‐positive human endometrial cancer cell proliferation through reducing the expression and activity of the Akt and ERα signalling pathway.^22^ However, the role of solasonine in gastric cancer remains to be investigated.

In this study, solasonine was found to remarkably inhibit the proliferation of human gastric cancer SGC‐7901 cells in a dose‐dependent manner in vitro, which was in agreement with previous reports.^17^, ^18^ The 24 h IC_50_ value of solasonine against SGC‐7901 cells was 18 μM, which was close to that of cisplatin (17.5 μM). Flow cytometry a comparable apoptotic rate in SGC‐7901 cells treated with 18 μM solasonine (40.4%) and 17.5 μM cisplatin for 24 h (44%), which further confirmed the comparative in vitro inhibition of these two agents against gastric cancer cells. However, solasonine and cisplatin showed diverse effects on the cell cycle of SGC‐7901 cells. In this study, flow cytometry showed that solasonine treatment mainly induced the cell cycle arrest at G2 phase in SGC‐7901 cells, while cisplatin predominantly arrested SGC‐7901 cells at G0/G1 phase. Cisplatin is reported to bind to DNA to inhibit DNA replication, thereby inducing cell cycle arrest at G0/G1 phase.^23^, ^24^ Till date, the mechanisms underlying solasonine‐induced cell cycle arrest at G2 phase remain unclear, and it is hypothesized that solasonine may block the tetraploid chromosomal division, which is similar to the mechanisms of actions of natural products such as actinomycin D and mitomycin C^25^, ^26^; however, the exact mechanisms for solasonine‐induced G2‐phase cell cycle arrest deserve further investigations.

Currently, there are three main apoptosis‐associated signalling pathways, including mitochondrial apoptotic pathway, cell death receptor pathway and endoplasmic reticulum stress pathway.^27^, ^28^, ^29^ Previous studies have demonstrated that solasonine induces the reactive oxygen species (ROS)‐mediated apoptosis through triggering the endoplasmic reticulum stress apoptotic pathway.^12^ In the current study, solasonine treatment significantly suppressed Bcl‐2 protein expression in SGC‐7901 cells, which was consistent with previous findings in HCC HepG2 cells and prostate cancer PC‐3 cells.^30^, ^31^ Bcl‐2 family proteins, a critical factor that determines the opening and closure of the mitochondrial permeability transition pore (mPTM), are a key protein that triggers the mitochondrial apoptotic pathway.^32^ Down‐regulation of Bcl‐2 protein expression causes mitochondrial damages and a reduction in mitochondrial Ca^2+^ concentrations, and this induces a rise in intracellular Ca^2+^ concentrations and a reduction in mitochondrial membrane potential, which activates Caspase‐3 and reduces the Bcl‐2/Bax ratio, thereby inducing the cell cycle arrest at M phase.^33^, ^34^, ^35^ Solasonine was found to reduce the level of both Bax and Bcl‐2 in gastric cancer cells simultaneously, and decreased the Bax/Bcl‐2 ratio (about 2.1 folds). It is therefore considered that solasonine induce gastric cancer cell apoptosis through the mitochondria‐mediated pathway.

Previous studies have shown that solasonine induces apoptosis of liver cancer, breast cancer and prostate cancer cells via the mitochondrial signalling pathway; however, there is little knowledge on the effects of solasonine on gastric cancer, and there is no knowledge pertaining to the in vivo activity of solasonine against gastric cancer until now. Since solasonine is major active ingredient of the medicinal herb *Solanum nigrum*, most studies pertaining to the pharmaceutical actions of solasonine have been performed by Chinese scientists. It has been shown that solasonine may increase the erythrocyte membrane fluidity to restore the immune functions of erythrocytes in tumour‐bearing mice, thereby resulting in antitumour activity.^36^ Zhang^18^ suggested that solasonine against the gastric cancer via modulation miR‐486‐5p/PI3KR1 axis.

In summary, the results of the present study demonstrate that solasonine shows a comparable inhibitory effect on the proliferation of human gastric cancer SGC‐7901 cells with cisplatin, and solasonine induces of SGC‐7901 cell apoptosis through triggering the endoplasmic reticulum stress pathway and the mitochondrial pathway. In addition, the cisplatin‐solasonine combination may improve the efficacy against gastric cancer. Our data indicate that solasonine may be a promising agent for the treatment of gastric cancer.

## AUTHOR CONTRIBUTION

**Tian‐chuan Li:** Investigation (equal); Resources (equal); Writing – original draft (equal). **Nai‐jie Chen:** Formal analysis (equal); Investigation (equal); Methodology (equal). **Yun‐ying Chen:** Investigation (equal); Project administration (equal); Software (equal). **Bing‐jin He:** Conceptualization (equal); Investigation (equal); Supervision (equal); Validation (equal); Writing – review & editing (equal). **feng zhi zhou:** Conceptualization (equal); Funding acquisition (equal); Supervision (equal); Validation (equal); Writing – review & editing (equal).

## CONFLICT OF INTEREST

The authors confirm that there are no conflicts of interest.

## Data Availability

The data are available on request from the authors.
